# Blood Pressure Variability After Acute Ischemic Stroke and Intracerebral Hemorrhage: Refining Its Definition, Intervention Opportunities, and Research Directions

**DOI:** 10.1007/s12028-025-02263-8

**Published:** 2025-05-06

**Authors:** David Z. Rose, Alejandro A. Rabinstein, May Kim-Tenser, Sergio D. Bergese, Gabriel V. Fontaine, Charles Kircher, Adnan I. Qureshi

**Affiliations:** 1 University of South Florida Health, Tampa, FL USA; 2 Mayo Clinic, Rochester, MN USA; 3 Keck Medicine of USC, Los Angeles, CA USA; 4 Stony Brook University, Stony Brook, NY USA; 5 Intermountain Health, Salt Lake City, UT USA; 6 Washington University School of Medicine in St. Louis, St. Louis, MO USA; 7 Zeenat Qureshi Stroke Institute and Department of Neurology, University of Missouri, Columbia, MO USA

## Abstract

Increased blood pressure variability (BPV) in the acute phases of cerebrovascular emergencies, such as acute ischemic stroke (AIS) and intracerebral hemorrhage (ICH), has been shown to result in worsened outcomes. Although several studies have reported this association, no consensus exists for specific BPV targets or a consistent, unified definition of BPV in AIS or ICH. Therefore, we convened the Blood Pressure Variability in Cerebrovascular Emergencies Consortia, consisting of a multidisciplinary group of experts in stroke, neurocritical care, perioperative medicine, emergency medicine, and clinical pharmacy to assess the clinical impact of BPV and to develop a working consensus on defining BPV, identifying interventions to mitigate negative outcomes from increased BPV, and laying the groundwork for BPV research concepts in the future. First, the Consortia proposed bifurcating systolic BPV (SBPV) into two distinct periods—SBPV_1_ and SBPV_2_. SBPV_1_ involves hyperacute management, when rapid and smooth blood pressure control is crucial. SBPV_2_, the plateauing phase, consists of a more gradual, maintenance-therapy slope. For both periods, enabling a “smooth” (SBPV_1_) and “sustained” (SBPV_2_) trajectory is likely ideal, but more phase-specific research is required to validate this concept. Secondly, Consortia proposed to calculate BPV by subtracting maximum and minimum systolic blood pressure over subsequent measurements because it represents the most clinically feasible option among many proposed equations in the literature. Third, for ICH, the Consortia preferred intravenous antihypertensive medication to reach BPV goals as fast, safe, and efficiently as possible, consistent with American Heart Association/American Stroke Association guidelines recommending “treatment regimens that limit BPV and achieve smooth, sustained blood pressure control.” For AIS, guidelines do not yet address BPV, but Consortia members proposed an algorithm with distinct SBPV goals based on time (as a function of stroke acuity), arterial subtype (large, medium, and small vessel), thrombolytic and/or thrombectomy status, and presenting SBP. As the understanding of BPV evolves, future research may build on and/or refine concepts proposed by this Consortia.

**Supplementary Information:**

The online version contains supplementary material available at 10.1007/s12028-025-02263-8.

## Introduction

For acute ischemic stroke (AIS) and intracerebral hemorrhage (ICH), research suggests that as blood pressure variability (BPV) increases, clinical outcomes worsen [1–6]. Higher BPV is associated with recurrent stroke [1], symptomatic intracranial hemorrhage after endovascular therapy [4], and increased disability and death at 90 days [2, 3, 5, 6]. Despite this association, no consensus exists for a definition of BPV in AIS, ICH, or for BPV targets. In 2022, the American Heart Association (AHA)/American Stroke Association (ASA) guidelines in spontaneous ICH recommended “maintaining smooth, sustained blood pressure control, avoiding peaks and large variability” to improve outcomes after ICH (class of recommendation: 2a; level of evidence: B, nonrandomized) [7]. For ICH associated with acute hypertensive response (systolic blood pressure [SBP] 150‒220 mm Hg), AHA/ASA guidelines recommended a target SBP of 140 mm Hg with maintenance SBP of 130‒150 mm Hg [7]. No recommendation is provided for baseline SBP > 220 mm Hg. The guideline also emphasizes expedited care, noting that blood pressure (BP) treatment within 2 h of ICH and reaching BP targets within an hour may improve functional outcome (class: 2a; level: C, limited data) [7]. The European Stroke Organisation acknowledges that benefits and risks of intensive BP lowering on outcomes remain uncertain after acute ICH [8]. In hyperacute ICH, the European Stroke Organisation recommends lowering SBP below 140 mm Hg to reduce hematoma expansion, but keeping it > 110 mm Hg and avoiding SBP drop > 90 mm Hg (quality of evidence: moderate; strength of recommendation: weak) [8].

For AIS, the 2019 AHA/ASA Guideline recommended maintaining BP < 180/105 mm Hg for ≥ 24 h after intravenous thrombolysis (IVT) but did not offer BPV recommendations [9]. Specific SBP goals after IVT were not provided from the cited Enhanced Control of Hypertension and Thrombolysis Stroke Study (ENCHANTED) trial, in which 90-day outcomes were similar for patients with SBP targets < 180 mm Hg versus 130–140 mm Hg [9, 10]. Thus, practice guidelines recommend “smooth” and “sustained” BP control in acute ICH, guideline recommendations for both ICH and AIS remain nonspecific [5, 7], and evidence supporting specific SBP targets is limited.

Because this BPV quandary remains unsolved, we convened the Blood Pressure Variability in Cerebrovascular Emergencies (B-PRECISE) Consortia, an invited group of seven experts (all authors of this article) in vascular neurology, neurocritical care, perioperative medicine, emergency medicine, and clinical pharmacy. We met in person before the International Stroke Conference in Phoenix, Arizona in February 2024. Follow-up meetings occurred virtually, after Consortia members reviewed and edited drafts of the proceedings. This consensus is based on the current (limited) literature and collective decades of multidisciplinary clinical experience. Discussion questions raised by the Consortia (Table 1) had the ultimate goals to: initiate evidence-based dialogue about BPV; stimulate future research strategies on post-stroke BPV; and, similar to “CODE ICH” standardizing ICH hyperacute treatment [11], recognize that post-stroke BPV management represents an unmet need ripe for standardization.

**Table 1:** Discussion questions at the Blood Pressure Variability in Cerebrovascular Emergencies Consortia meeting

Question
1. What is the clinical impact of BPV on outcomes in AIS and ICH?
2. How should BPV be practically defined?
3. What is a practical way to calculate BPV at the bedside?
4. Which therapeutic interventions may help reduce BPV and potentially mitigate negative outcomes?
5. Based on expert opinion, what general BPV targets should be proposed for AIS and ICH?

AIS, acute ischemic stroke, BPV, blood pressure variability, ICH, intracerebral hemorrhage

### BPV and Stroke Outcomes

Evidence of a relationship between BPV and poor functional outcomes derives mostly from prospective cohort studies and post hoc analyses of randomized controlled trials (Table 2 and Supplement) [12–14]. The Intensive Blood Pressure Reduction in Acute Cerebral Hemorrhage Trial 2 (INTERACT2) and Antihypertensive Treatment of Acute Cerebral Hemorrhage 2 (ATACH-2) failed to demonstrate significant associations between intensive SBP control and 90-day functional outcomes or death after ICH, but suggested that greater BPV may result in neurological deterioration and worse functional outcomes [15, 16].

**Table 2:** Select studies evaluating BPV in ICH (above) and AIS (below)

Study	Design	Patient population	BPV index	Outcomes measured	BP interventions	Outcomes
ICH						
AINS, 2024 [17]	Prospective, observational cohort study	ICH (*n* = 312)	SD	Hematoma expansion END rate (increase in NIHSS score ≥ 4 or death at 24 h) mRS shift Independent ambulation at 90 days (mRS ≤ 3) Functional independence at 90 days (mRS ≤ 2)	Single IV bolus, followed by continuous IV infusion First-line urapidil and second-line labetalol were recommended	Higher 24-h SBPV was: Not related to hematoma expansion Associated with higher END rate and 90-day mRS scores
INTERACT2 and ATACH-2, 2019 [18]	Preplanned pooled analysis of RCTs	ICH (*n* = 3829)	SD	Functional status (mRS) Good outcome (mRS ≤ 3) Functional independence (mRS ≤ 2) Increase hematoma volume Death within 90 days	INTERACT2: Intensive vs. guideline- recommended treatment (variety of agents permitted) ATACH-2: Nicardipine, then labetalol (where available)	Abnormal BPV associated with functional status, mRS, functional independence, hematoma expansion, death
EnRICH, 2019 [19]	Prospective cohort study	ICH (*n* = 556)	SD	mRS at 90 days	Nicardapine, labetalol, esmolol, metoprolol	Patients with mRS 4–6 and those who died in-hospital or within 30- or 90-days after discharge were more likely to have high systolic BPV
ATACH-2, 2018 [3, 16]	Post hoc analysis of RCT	ICH (n = 1,000)	SD, CV, ARV, SV, rSD	Unfavorable neurological outcome (mRS ≥ 3) at 90 days	Nicardipine, then labetalol (where available)	Abnormal BPV associated with unfavorable neurological outcome at 90 days
FAST-MAG, 2018 [6]	Post hoc analysis of phase 3 RCT	ICH (*n* = 386)	SD, CV*, SV, max − min SBP	Poor 3-month outcome (mRS ≥ 3)	Per guidelines and physician’s discretion (specific agents not provided)	Abnormal BPV associated with poor 3- month outcomes
INTERACT2, 2014 [5]	Post hoc analysis of open-label RCT	ICH (*n* = 2839)	SD	mRS ≥ 3 at 90 days Ordinal shift in mRS at 90 days	Intensive vs. guideline- recommended treatment (variety of agents permitted)	Abnormal BPV associated with mRS ≥ 3 and ordinal shift in mRS at 90 days
SAMURAI, 2012 [20, 21]	Prospective cohort study	ICH (n = 205)	SD, SV	Hematoma expansion Decrease ≥ 2 in Glasgow Coma scale Increase ≥ 4 in NIHSS Unfavorable outcome (mRS ≥ 4)	Nicardipine	Abnormal BPV associated with unfavorable outcome
AIS						
BP TARGET, 2022 [22]	Post hoc analysis of RCT	AIS (n = 290)	SD, CV, SV, max − min SBP, TR	Favorable functional outcome (mRS 0–2) ICH rate at 24 h	Endovascular thrombectomy	Inconsistent associations between abnormal BPV and outcomes, depending on BPV index used and measurement of SBP vs. DBP
BEST, 2020 [2]	Post hoc analysis of prospective cohort study	AIS (*n* = 443)	SD, CV, ARV, SV, rSD	Poor outcome at 90 days (mRS ≥ 3)	Endovascular thrombectomy	All abnormal BPV measures associated with poor outcome or death

Summary of key studies that evaluated BPV in ICH and AIS

ARV, average real variability, BEST, blood pressure after endovascular therapy for ischemic stroke, BP, blood pressure; BPV, blood pressure variability, CV, coefficient of variation; END, early neurologic deterioration, FAST-MAG, field administration of stroke therapy-magnesium, ICH, intracranial hemorrhage, max, maximum, min, minimum, mRS, modified Rankin Scale, NIHSS, National Institutes of Health Stroke Scale, RCT, randomized controlled trial, rSD, residual standard deviation, SBP, systolic blood pressure, SBPV, systolic blood pressure variability, SD, standard deviation, SV, successive variation, TR, time rate

### Proposing a Practical Definition of BPV

**Fig. 1:**
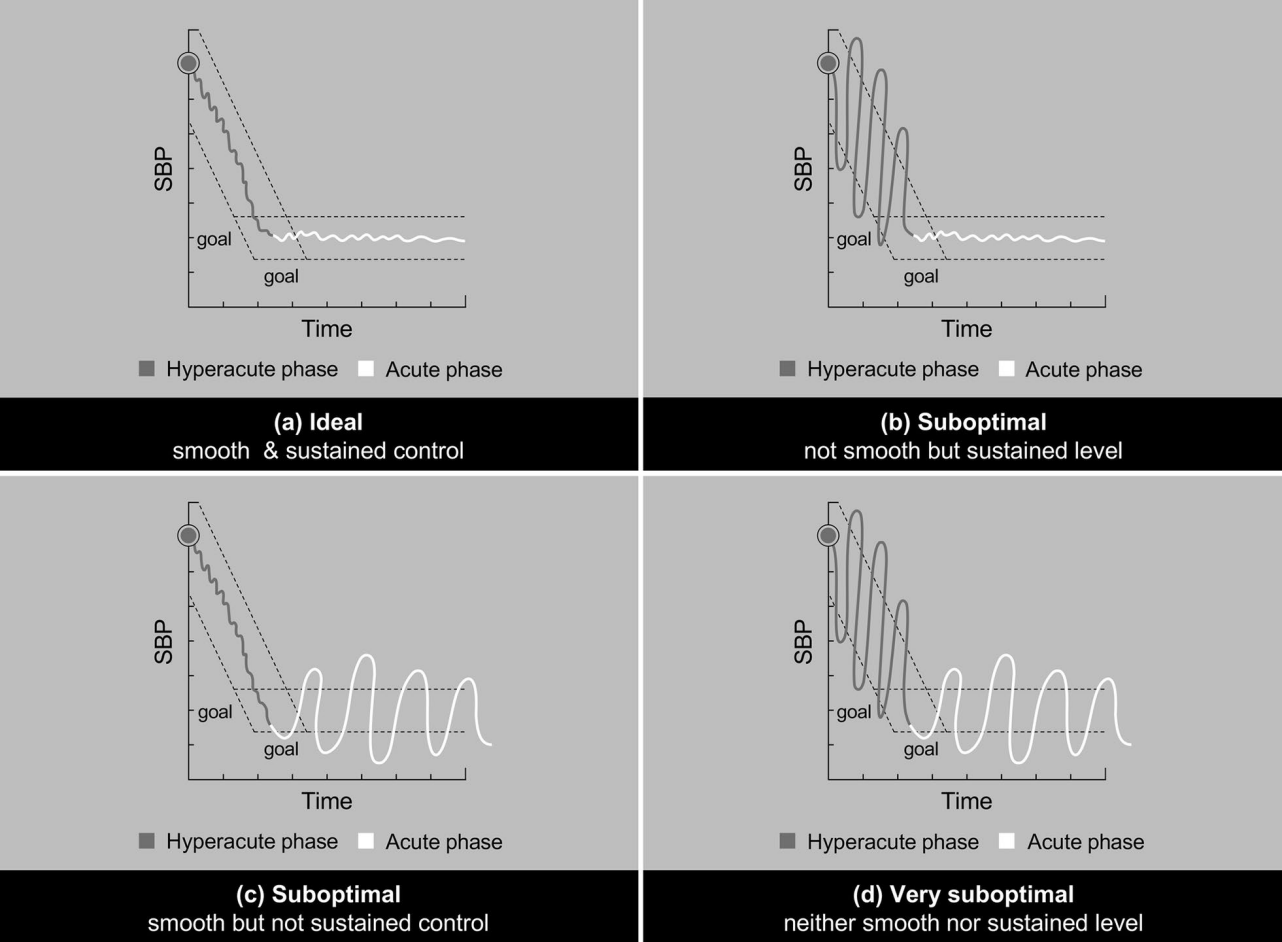
SBPV Scenarios with Varying Levels of “Smooth” and “Sustained” Control. The concept of distinct periods of SBPV over time: **a** ideal “smooth” and “sustained” SBP; **b**: nonsmooth but sustained BPV; **c**: smooth but nonsustained BPV; and **d** neither smooth landing nor sustained control, representing a very suboptimal pattern

Studies that examined BPV have used various definitions of BPV and a panoply of BPV endpoints (Table 2). Knowledge gaps exist because: (1) BPV is not yet practically defined or routinely assessed; (2) BPV goals may require tailoring to specific clinical scenarios; and (3) pharmacological options to minimize BPV have not been thoroughly, formally evaluated. Therefore, the Consortia proposed a practical definition of BPV, focusing on BPV timing and bifurcating SBP variability (SBPV) into two segments (Fig. 1):

1. The hyperacute period, lasting from stroke onset to target SBP attainment (SBPV_1_), and
2. The acute period, beginning at target SBP attainment and ideally maintained as a sustained plateau (SBPV_2_)

There is a precedent for this categorization. Post hoc analyses of INTERACT2 and ATACH-2 only examined SBPV_2_ to limit bias from study interventions eliciting rapid BP reductions. ATACH-2 investigators did not include BP readings at hours 0–2 after randomization [3], and INTERACT2 BPV analyses excluded BP readings during the first hour after treatment [5]. Conversely, smaller observational studies have included all BP measurements [12, 23].

When managing BPV, this Consortia proposes considering SBPV_1_ and SBPV_2_ separately, to address distinct SBPV goals in the hyperacute and acute settings. Consortia proposes that it is ideal to minimize BPV in a smooth (SBPV_1_) and sustained (SBPV_2_) manner like an airplane landing (Fig. 1a) or hypothermia protocol after cardiac arrest. Instead, excessive variations in descent and plateau may result in suboptimal outcomes (Fig. 1b–d) and increase the risk of neurologic deterioration hematoma expansion, and/or watershed strokes. However, these concepts require validation in clinical trials.

### Practical BPV Calculation

Many statistical methods have been crafted to measure BPV, including SD, residual SD, coefficient of variation (CV), successive variation (SV), functional successive variation (FSV), average real variability (ARV), and the difference between the maximum and minimum SBP (max–min) [1–4, 23–26]. Although some studies employed one or two of these equations [23] many implemented multiple BPV measures (Table 2) [2–4, 27, 28]. This surplus has resulted in no clear standard. To inform these measurements, using an arterial line to continuously monitor SBP will likely provide the most accurate readings and capture sufficient data points, but invasive monitoring is challenging to implement clinically.

Eventually, artificial intelligence (AI) systems may rapidly calculate complex BPV equations in real-time, and translate data into actionable interventions, similar to current AI progress with neuroradiology and neurocardiology [29]. Presently, however, electronic medical records (EMR) are unable to adjust to the dynamic nature of BP over time, typically only recording intermittent, hand-typed BP data [30]. Therefore, we propose using the utilitarian SBP range (max–min) to yield quick and meaningful parameters. This approach, while basic, is supported by retrospective data reporting that SBP ranges are associated with 90-day modified Rankin Scale (mRS) and mortality in both AIS [4, 25] and ICH [3, 5, 6]. Additional testing of BPV measurements and how to incorporate them into clinical practice, especially into EMR with AI, is needed.

### Treating Acute Hypertension in Stroke

For AIS, SBP > 185 mm Hg and DBP > 110 mm Hg are IVT relative contraindications, but timely reduction may qualify patients. Patients without IVT receive a “permissive hypertension” strategy (i.e., lowering from > 220 mm Hg to < 220 mm Hg). Although initial SBP control is usually achievable, frequent titration and second/third antihypertensives for “rebound hypertension” are often needed, increasing resource utilization and decreasing efficiencies [31]. After reaching target SBP, minimizing SBPV_2_ is crucial [18, 31]. Emerging literature shows a relationship between BPV and functional outcomes, highlighting the unmet need for prompt and predictable treatments that minimize SBPV in a “smooth and sustained” manner [32–34].

Table 3 summarizes classes of antihypertensives commonly used in hyperacute and acute stroke settings. Bolus dosing, although effective at reducing SBP quickly, carries a risk of increased BPV after administration [34]. Intermediate-to-long-acting antihypertensives may yield smooth reductions in BP, but their longer half-lives preclude timely dose modifications to limit SBPV_2_ if/when hypotension or overcorrecting occurs [33].

**Table 3:** Summary of drug classes available for intervention in the acute setting

Administration	Overview of antihypertensives		
	Drug class	Onset (min)	Half-life
*IV infusion*			
Rapid acting (sec)	Third-generation dihydropyridine calcium channel blocker* [41, 42]; direct-acting vasodilator [33, 43]	2–4; < 2	Early (alpha) half-life: 1 min, terminal (gamma) half-life: 15 min; 2 min
Intermediate acting (min)	Selective beta1 adrenergic receptor blocker [42, 44]; second-generation dihydropyridine calcium channel blocker* [33, 42, 45]; selective alpha1 and nonselective beta adrenergic receptor blocker^†^ [42, 46]	2–10; 5–10; 5–15	Distribution half-life: 2 min, elimination half-life: 9 min; early (alpha) half-life: 3 min, intermediate (beta) half-life: 45 min, terminal (gamma) half-life: 14.4 h; 5.5 h
Bolus	Selective alpha1 and nonselective beta adrenergic receptor blocker^†^ [42, 46]; vasodilator [42, 47]; angiotensin-converting enzyme inhibitor [42, 48]	2–5; 5–20; 15–30	5.5 h; 2–8 h depending on slow/fast acetylator; 11 h

^*^Dihyrdopyridine calcium channel blockers are available in rapid- and intermediate-acting formulations

^†^Some adrenergic blocking agents may be administered via intravenous infusion or as a bolus dose

To limit BPV, ATACH-2 investigators proposed preferential use of a monotherapeutic, short-acting antihypertensive infusion [3]. In a systematic review and meta-analysis, dihydropyridine calcium channel blockers (CCBs) and nonloop diuretics reduced interindividual SBPV more than angiotensin-converting enzyme inhibitors, angiotensin receptor blockers, or beta-blockers [35]. These data identify drug classes that may have consistent effects on SBPV in the hyperacute/acute settings; for example, ultra-short-acting nondihydropyridine CCBs reach prespecified, tight SBP target ranges in 96.9% of patients within 30 min [36]. High-fidelity, high-predictability agents such as the latest-generation CCBs, have been shown to keep SBP within target range (limiting BPV) and limit multiple titrations [36]. Moreover, reducing median time for initial parenteral antihypertensive dose-to-goal (DTG) may result in improved door-to-needle (DTN) thrombolytic times [37] and theoretically less infarcted brain tissue. Future research may focus on prehospital [38] and emergency department DTG and DTN opportunities.

Nitrous vasodilators reach target SBP ranges within 2 min [33, 39] but may significantly increase intracranial pressure, reduce CPP [33, 40] and BP fluctuations are common (only a 69% time within target BP range for ICH) [39]. Nitrous agents therefore are typically avoided for ICH/AIS with cerebral edema, or intracranial mass lesions.

Supporting ATACH-2 investigators’ hypothesis [3] and consistent with AHA/ASA guidelines recommending treatments “that limit BPV and achieve smooth, sustained [BP] control” [7], the Consortia favor shortest-acting intravenous antihypertensive infusions to reach SBP goals while minimizing BPV goals. More research is necessary to determine the optimal antihypertensive regimen in these patients.

### Expert Opinion: Proposed BPV Targets

Tables 4 and 5 outlines a general approach to defining target SBPV ranges for AIS and ICH, based on intervention status and presenting SBP. These targets represent our expert opinion on SBPV goals and require validation. The scientific rationale for these targets is hypothetical, presumably from vasoplegic arteries that may be prone or sensitive to BPV in the hyperacute phase. For AIS, a reasonable starting point is indentifying SBPV targets by vessel recanalization and IVT/endovascular therapy status (Table 4). For patients with AIS and large-vessel occlusion (LVO) or medium-vessel occlusion (MeVO) who become fully revascularized (TICI 3), there is no flow-dependent deficit. Therefore, fluctuations of < 40 mm Hg during SBPV_1_ and < 30 mm Hg during SBPV_2_ may be acceptable. By contrast, in patients with LVO or MeVO and minimal/no revascularization (TICI 0–1), any precipitous SBP drop can exacerbate cerebral ischemia. These patients require particularly cautious SBP lowering and keeping SBPV_1_ and SBPV_2_ < 20 mm Hg would be advisable. For patients with partial reperfusion (TICI 2a–2c), the proposed SBPV_1_ target lies between revascularized and nonrevascularized targets, whereas SBPV_2_ may need to be < 20 mm Hg.

**Table 4:** Proposed SBPV targets in AIS

Arterial subtype	Proposed variability range (mm Hg)*^†^	
	**Hyperacute phase (< 24 h)**	**Acute phase (24–72 h)**
LVO or MeVO recanalization and/or thrombolytic status		
TICI 3 ± IVT	< 40	< 30
TICI 2a–2c ± IVT	< 30	< 20
TICI 0–1 ± IVT	< 20	< 20
SVO recanalization and/or thrombolytic status		
IVT	< 20	< 20
No IVT	< 40	< 30

IVT, intravenous thrombolysis, LVO, large vessel occlusion, MeVo, medium-vessel occlusion, SVO, small vessel occlusion, SBPV, systolic blood pressure variability, TICI, thrombolysis in cerebral infarction scale

^*^Therapy-based and/or spontaneous, incremental reduction in variability is expected with longer time interval from symptom onset

^†^Expert opinion only and warrants validation in clinical research studies

**Table 5:** Proposed SBPV targets in ICH

Presenting SBP (mm Hg)	Proposed variability range (mm Hg)*^†^	
	Hyperacute phase (< 12 h)	Acute phase (12–72 h)
> 220	< 20	< 10
180–220	< 30	< 20
140–180	< 40	< 20
< 140	< 40	< 30

SBP, systolic blood pressure; SBPV, systolic blood pressure variability

^*^Therapy-based and/or spontaneous, incremental reduction in variability is expected with longer time interval from symptom onset

^†^Expert opinion only and warrants validation in clinical research studies

For ICH, the Consortia proposed SBPV targets dependent on presenting SBP (Table 5). For patients with SBP > 220 mm Hg, tight SBPV_1_ and SBPV_2_ goals are desirable because these extremely hypertensive individuals may be at greatest risk of ischemia with major BP drops. For patients with SBP 180–220 mm Hg, we suggest an SBPV_1_ goal of < 30 mm Hg and a tighter goal in SBPV_2_. For patients with SBP 140–180 mm Hg, a relatively loose SBPV_1_ of < 40 mm Hg and SBPV_2_ of < 20 mm Hg may be permissible. Lastly, normotensive patients may tolerate wider SBPV ranges in both phases.

## Discussion and Future Directions

Studies of BPV after AIS/ICH show that higher SBPV portends worse clinical outcomes, including recurrent stroke [1] and increased disability and death at 90 days [2, 3, 5, 6]. However, currently no consensus exists for specific BPV targets or management strategies. Leveraging the limited data available, along with clinical experience, the Consortia proposed SBPV targets for AIS and ICH, based on intervention status and presenting SBP. Our proposals (Table 6) represent expert opinions only but will hopefully spur discussion and research efforts to validate these targets.

**Table 6:** Multidisciplinary expert consensus for evaluating and managing BPV in clinical practice

Topic	Multidisciplinary panel consensus for implementation in clinical practice			
Calculating and monitoring BP	Calculating BPV: In clinical practice, measure BPV by recording an SBP range (maximum − minimum) In clinical research, testing a variety of BPV measures is warranted			
BPV management	Using intravenous antihypertensives may enable prompt, but predictable attainment of target SBP that can be sustained over time in both ICH and AIS Minimize the need for dynamic BP control and frequent dose adjustments To limit BP fluctuations below the minimum threshold desirable, select antihypertensive therapies with a low frequency of hypotension			
Proposed SBPV targets in AIS	Arterial subtype	Recanalization and/or thrombolytic status	Proposed variability range (mm Hg)*^†^	
			Hyperacute phase (< 24 h)	Acute phase (24–72 h)
	LVO or MeVO	TICI 3 ± IVT	< 40	< 30
		TICI 2a–2c ± IVT	< 30	< 20
		TICI 0–1 ± IVT	< 20	< 20
	SVO	IVT	< 20	< 20
		No IVT	< 40	< 30
Proposed SBPV targets in ICH	Presenting SBP (mm Hg)		Proposed variability range (mm Hg)*^†^	
			Hyperacute phase (< 12 h)	Acute phase (12–72 h)
	> 220		< 20	< 10
	180–220		< 30	< 20
	140–180		< 40	< 20
	< 140		< 40	< 30

^*^Therapy-based and/or spontaneous, incremental reduction in variability is expected with longer time interval from symptom onset

^†^Expert opinion only and warrants validation in clinical research studies

The 2022 AHA/ASA ICH guideline recommends a SBP of 130‒150 mm Hg, with a goal of maintaining it in a 20 mm Hg range (class IIb, level B-Randomized). The 2013 Acute Hypertension and Intracerebral Hemorrhage with Intravenous Clevidipine Treatment (ACCELERATE) trial also prespecified a 20 mm Hg target SBP range (140‒160 mm Hg) for ICH [36] Median time to target SBP was 5.5 min in ACCELERATE, which nearly all patients achieved by 30 min, and the SD of SBP (essentially SBPV_2_) narrowed within minutes [36]. This indicates that for supratentorial ICH due to hypertensive emergency, strict SBPV targets are possible within 30 min. Tighter SBPV goals are reinforced by data from ATACH-2, where the SD of SBP (from 2 to 24 h) was 15.1 ± 5.8 mm Hg versus 13.7 ± 4.4 mm Hg (*P* < 0.001) in patients with 90-day mRS 3–6 and mRS < 3, respectively [3]. This difference persisted from days 2 to 7, when SD was 25.4 ± 8.6 mm Hg versus 21.1 ± 7.8 mm Hg in the poor and good outcome groups, respectively (*P* < 0.001) [3]. Similarly, in INTERACT2, SD of SBP was significantly associated with worse functional outcome [3] (≥ 20.5 mm Hg vs. < 8.4 mm Hg in the highest and lowest quintiles) [5]. Although optimal ranges for SBPV after AIS remains unknown, we hypothesize that tighter SBPV for certain scenarios may result in better outcomes.

Limitations of this article include our inability to propose targets for every clinical scenario due to space constraints, plus insufficient data to support such recommendations. For example, patients with secondary hypertension due to endocrine disorders (pheochromocytoma, hyperaldosteronemia, etc.), high-risk features (e.g., spot sign, swirl sign, coagulopathy), severely uncontrolled “malignant” hypertension, severe cervical or intracranial arterial stenosis, aneurysmal subarachnoid hemorrhage, and other pathologies may have different SBPV targets or require intervention at alternative SBPV thresholds. Those scenarios, although clinically relevant, are beyond the scope of this inaugural Consortia consensus. A critical part of future research will be to refine actionable SBPV limits for various clinical scenarios.

Optimal treatment strategies for achieving the proposed SBPV targets remain unknown. Goals of therapy include maintaining a steady SBP and minimizing the need for dose adjustments during SBPV_2_ [31, 33]. With these goals in mind and building on ATACH-2 investigators’ hypothesis [3] and AHA/ASA guideline recommendations [7] the Consortia proposes using intravenous antihypertensives that smoothly and predictably reach and maintain target SBP levels. Studies are needed to test intravenous antihypertensives in various stroke settings, including prehospital [38]. As our understanding of BPV evolves, future data will help modify the concepts we have proposed.

Other limitations include varied definitions for “poor outcomes” in BPV research. INTERACT2, ATACH-2, and others defined poor outcomes as mRS 3–6 at 90 days [2, 3, 5, 6] while other studies defined “unfavorable” as mRS 4–6 at discharge [24], a 1-point increase in mRS [27], recurrent stroke [1], early neurologic deterioration [49], and death [1]. Another limitation is the unknown relative effects of SBPV_1_ and SBPV_2_ on outcomes. In INTERACT2, associations between BPV and death or major disability were only significant when early SBP measurements were excluded [5]. Ideally, future studies will analyze each SBPV phase. Studies are also needed to evaluate the relative merits of various BPV calculation methods in clinical practice. Of BPV measures studied, SD seems most commonly employed, but its utility at the bedside remains unclear [2–5, 12, 25, 27]. More sophisticated BPV calculations (e.g., SV, FSV, ARV) have been proposed but are currently unwieldy to implement outside of research studies. Nevertheless, AI applied to EMR shows promise [29]. Finally, studies should also examine whether frequent monitoring and early SBPV interventions are warranted for patients with risk factors for excessive SBPV (e.g., SBP > 220 mm Hg, older age, diabetes, and higher National Institutes of Health Stroke Scale and/or Glasgow Coma Scale scores), as identified in the Efficient Resource Utilization for Patients with Intracerebral Hemorrhage (EnRICH) study [19].

In summary, there is paucity of robust clinical trials assessing SBPV in hyperacute/acute AIS and ICH. Drawing definitive conclusions is challenging due to heterogeneity of BPV descriptions, calculations, and management in the literature. Whether certain antihypertensives to reduce BPV after AIS/ICH affects outcomes remains unknown. The goal of this document, therefore, is to make BPV a practical concept that can be tested as a target for intervention after AIS and ICH.

## Supplementary Information

Below is the link to the electronic supplementary material.

Supplementary file1 (DOCX 85 kb)
