# Supplementary material for: Blood Pressure Variability After Acute Ischemic Stroke and Intracerebral Hemorrhage: Refining Its Definition, Intervention Opportunities, and Research Directions
Source: Neurocrit Care. 2025 May 6;43(2):368–77. doi: 10.1007/s12028-025-02263-8 (PMC12436555; doi:10.1007/s12028-025-02263-8)
Supplement: Supplementary file 1 — Supplementary file1 (DOCX 85 kb) [file 12028_2025_2263_MOESM1_ESM.docx]

**SUPPLEMENTARY INFORMATION TO:**

**Blood Pressure Variability after Acute Ischemic Stroke and Intracerebral Hemorrhage:**

**Refining its Definition, Role as a Target for Intervention, and Research Opportunities**

Multidisciplinary Expert Consensus: The Blood Pressure Variability in Cerebrovascular Emergencies (B-PRECISE) Consortia

David Z. Rose, MD; Alejandro A. Rabinstein, MD; May Kim-Tenser, MD MHA; Sergio D. Bergese, MD FASA;

Gabriel V. Fontaine, PharmD MBA; Charles Kircher, MD MBA; Adnan I. Qureshi, MD

**Effects of BPV on Outcomes: Supplemental Summary**

Evidence of a relationship between wider BPV and poor functional outcomes in the acute setting derives mostly from prospective cohort studies and post-hoc analyses of randomized controlled trials. [^1–3^](https://sciwheel.com/work/citation?ids=16004747,16004797,16332840&pre=&pre=&pre=&suf=&suf=&suf=&sa=0,0,0&dbf=0&dbf=0&dbf=0) A summary of these studies is provided here.

BPV and Outcomes in ICH

Renewed interest in BPV emerged after the INTERACT2 and ATACH-2 trials failed to demonstrate significant associations between intensive control of SBP and 90-day functional outcomes or death, potentially due to other contributing factors, such as BPV.[^4,5^](https://sciwheel.com/work/citation?ids=308471,1508753&pre=&pre=&suf=&suf=&sa=0,0&dbf=0&dbf=0) In the INTERACT2 post hoc analysis, the SD of SBP during the first 24 hours and on days 2 through 7 was significantly and linearly associated with poor outcomes on the 90-day mRS.[^6^](https://sciwheel.com/work/citation?ids=1529596&pre=&suf=&sa=0&dbf=0) Similarly, the post hoc analysis of the ATACH-2 trial reported that all measures of BPV, but not mean SBP, in both the acute and subacute periods were significantly associated with worse neurologic outcomes on the 90-day mRS.[^7^](https://sciwheel.com/work/citation?ids=16004741&pre=&suf=&sa=0&dbf=0) A 2019 pooled analysis of INTERACT2 and ATACH-2 identified that SD of SBP was significantly associated with death within 90 days, and conversely, patients with lower SBPV had better outcomes, including mRS 0-3 at 90 days and a favorable shift on the mRS.[^8^](https://sciwheel.com/work/citation?ids=9765978&pre=&suf=&sa=0&dbf=0) Other studies, including the FAST-MAG study and SAMURAI-ICH have reported similar results.[^9^](https://sciwheel.com/work/citation?ids=6310352&pre=&suf=&sa=0&dbf=0)^,^[^10^](https://sciwheel.com/work/citation?ids=16187929&pre=&suf=&sa=0&dbf=0)

**No Association Between SBPV and Hematoma Expansion or Between DBP and Functional Outcomes**

While higher BPV has consistently been linked to disability and death, the association may be unrelated to hematoma expansion and unaffected by mean arterial pressure (MAP) or DBP variability. Analyses of INTERACT2 and the FAST-MAG study found no association between BPV (SD and CV) and hematoma expansion after ICH.[^6,11^](https://sciwheel.com/work/citation?ids=16004695,1529596&pre=&pre=&suf=&suf=&sa=0,0&dbf=0&dbf=0) Similar outcomes were reported in a retrospective study of 1119 patients admitted to 2 health care systems over a span of 10 years.[^12^](https://sciwheel.com/work/citation?ids=16004705&pre=&suf=&sa=0&dbf=0) It demonstrated associations between SBPV and in-hospital outcomes (mRS at discharge) post-ICH, however, there was no link between BPV and hematoma expansion.[^12^](https://sciwheel.com/work/citation?ids=16004705&pre=&suf=&sa=0&dbf=0) In the same study, only SBPV was associated with outcomes, not diastolic BPV or MAP.[^12^](https://sciwheel.com/work/citation?ids=16004705&pre=&suf=&sa=0&dbf=0) Other studies have reported a similar lack of association between diastolic BPV and outcomes in the acute setting, but the relationship between fluctuations in MAP and outcomes is less clear.[^6,13^](https://sciwheel.com/work/citation?ids=8993007,1529596&pre=&pre=&suf=&suf=&sa=0,0&dbf=0&dbf=0)

BPV and Outcomes in AIS

The detrimental effects of wider BPV on outcomes have also been demonstrated in AIS. A meta-analysis of trials examining SBPV after EVT found that the SD, CV, and SV were associated with lower odds of achieving mRS ≤2 at 90 days, while variation independent of the mean SBP was not.[^1^](https://sciwheel.com/work/citation?ids=16004747&pre=&suf=&sa=0&dbf=0) Included in the meta-analysis was a post hoc analysis of the BEST study, in which all measures of BPV were significantly higher in patients with poor outcomes, SBP measures of BPV had a more pronounced association with functional outcome than DBP, and only SBP measures (not DBP) were associated with 90-day mortality.[^14^](https://sciwheel.com/work/citation?ids=9425455&pre=&suf=&sa=0&dbf=0) A retrospective multicenter international study at 10 comprehensive stroke centers in 2019 reported that greater BPV after EVT with successful revascularization was associated with higher likelihood of sICH, need for hemicraniectomy, and mortality.[^13^](https://sciwheel.com/work/citation?ids=8993007&pre=&suf=&sa=0&dbf=0)

After intravenous thrombolysis (IVT), one study from 2022 showed that the highest quintile of SBPV (measured by ARV) was associated with a higher risk of recurrent stroke and all-cause death within 72 hours.[^15^](https://sciwheel.com/work/citation?ids=16004782&pre=&suf=&sa=0&dbf=0) Similarly, a relationship between BPV and poor outcomes after AIS was reported in smaller studies and retrospective analyses of BPV after reperfusion therapy.[^13,16–19^](https://sciwheel.com/work/citation?ids=16034109,16034110,16034112,8993007,16004781&pre=&pre=&pre=&pre=&pre=&suf=&suf=&suf=&suf=&suf=&sa=0,0,0,0,0&dbf=0&dbf=0&dbf=0&dbf=0&dbf=0) Prospective data demonstrating causality in this relationship are needed.

**References:**

[1.    Nepal G, Shrestha GS, Shing YK, Muha A, Bhagat R. Systolic blood pressure variability following endovascular thrombectomy and clinical outcome in acute ischemic stroke: A meta-analysis. *Acta Neurol Scand*. 2021;144(4):343-354. doi:10.1111/ane.13480](https://sciwheel.com/work/bibliography/16004747)

[2.    Andalib S, Lattanzi S, Di Napoli M, et al. Blood pressure variability: A new predicting factor for clinical outcomes of intracerebral hemorrhage. *J Stroke Cerebrovasc Dis*. 2020;29(12):105340. doi:10.1016/j.jstrokecerebrovasdis.2020.105340](https://sciwheel.com/work/bibliography/16004797)

[3.    Zompola C, Palaiodimou L, Voumvourakis K, et al. Blood pressure variability in acute stroke: A narrative review. *J Clin Med*. 2024;13(7). doi:10.3390/jcm13071981](https://sciwheel.com/work/bibliography/16332840)

[4.    Anderson CS, Heeley E, Huang Y, et al. Rapid blood-pressure lowering in patients with acute intracerebral hemorrhage. *N Engl J Med*. 2013;368(25):2355-2365. doi:10.1056/NEJMoa1214609](https://sciwheel.com/work/bibliography/308471)

[5.    Qureshi AI, Palesch YY, Barsan WG, et al. Intensive Blood-Pressure Lowering in Patients with Acute Cerebral Hemorrhage. *N Engl J Med*. 2016;375(11):1033-1043. doi:10.1056/NEJMoa1603460](https://sciwheel.com/work/bibliography/1508753)

[6.    Manning L, Hirakawa Y, Arima H, et al. Blood pressure variability and outcome after acute intracerebral haemorrhage: a post-hoc analysis of INTERACT2, a randomised controlled trial. *Lancet Neurol*. 2014;13(4):364-373. doi:10.1016/S1474-4422(14)70018-3](https://sciwheel.com/work/bibliography/1529596)

[7.    de Havenon A, Majersik JJ, Stoddard G, et al. Increased blood pressure variability contributes to worse outcome after intracerebral hemorrhage. *Stroke*. 2018;49(8):1981-1984. doi:10.1161/STROKEAHA.118.022133](https://sciwheel.com/work/bibliography/16004741)

[8.    Moullaali TJ, Wang X, Martin RH, et al. Blood pressure control and clinical outcomes in acute intracerebral haemorrhage: a preplanned pooled analysis of individual participant data. *Lancet Neurol*. 2019;18(9):857-864. doi:10.1016/S1474-4422(19)30196-6](https://sciwheel.com/work/bibliography/9765978)

[9.    Chung P-W, Kim J-T, Sanossian N, et al. Association between hyperacute stage blood pressure variability and outcome in patients with spontaneous intracerebral hemorrhage. *Stroke*. 2018;49(2):348-354. doi:10.1161/STROKEAHA.117.017701](https://sciwheel.com/work/bibliography/6310352)

[10.   Tanaka E, Koga M, Kobayashi J, et al. Blood pressure variability on antihypertensive therapy in acute intracerebral hemorrhage: the Stroke Acute Management with Urgent Risk-factor Assessment and Improvement-intracerebral hemorrhage study. *Stroke*. 2014;45(8):2275-2279. doi:10.1161/STROKEAHA.114.005420](https://sciwheel.com/work/bibliography/16187929)

[11.   Oh DM, Shkirkova K, Poblete RA, et al. Association Between Hyperacute Blood Pressure Variability and Hematoma Expansion After Intracerebral Hemorrhage: Secondary Analysis of the FAST-MAG Database. *Neurocrit Care*. 2023;38(2):356-364. doi:10.1007/s12028-022-01657-2](https://sciwheel.com/work/bibliography/16004695)

[12.   Divani AA, Liu X, Di Napoli M, et al. Blood Pressure Variability Predicts Poor In-Hospital Outcome in Spontaneous Intracerebral Hemorrhage. *Stroke*. 2019;50(8):2023-2029. doi:10.1161/STROKEAHA.119.025514](https://sciwheel.com/work/bibliography/16004705)

[13.   Anadani M, Orabi MY, Alawieh A, et al. Blood pressure and outcome after mechanical thrombectomy with successful revascularization. *Stroke*. 2019;50(9):2448-2454. doi:10.1161/STROKEAHA.118.024687](https://sciwheel.com/work/bibliography/8993007)

[14.   Mistry EA, Mehta T, Mistry A, et al. Blood pressure variability and neurologic outcome after endovascular thrombectomy: A secondary analysis of the BEST study. *Stroke*. 2020;51(2):511-518. doi:10.1161/STROKEAHA.119.027549](https://sciwheel.com/work/bibliography/9425455)

[15.   He M, Wang H, Tang Y, et al. Blood pressure undulation of peripheral thrombolysis period in acute ischemic stroke is associated with prognosis. *J Hypertens*. 2022;40(4):749-757. doi:10.1097/HJH.0000000000003070](https://sciwheel.com/work/bibliography/16004782)

[16.   Bennett AE, Wilder MJ, McNally JS, et al. Increased blood pressure variability after endovascular thrombectomy for acute stroke is associated with worse clinical outcome. *J Neurointerv Surg*. 2018;10(9):823-827. doi:10.1136/neurintsurg-2017-013473](https://sciwheel.com/work/bibliography/16034109)

[17.   Cho BH, Kim JT, Lee JS, et al. Associations of various blood pressure parameters with functional outcomes after endovascular thrombectomy in acute ischaemic stroke. *Eur J Neurol*. 2019;26(7):1019-1027. doi:10.1111/ene.13951](https://sciwheel.com/work/bibliography/16034110)

[18.   Kim TJ, Park H-K, Kim J-M, et al. Blood pressure variability and hemorrhagic transformation in patients with successful recanalization after endovascular recanalization therapy: A retrospective observational study. *Ann Neurol*. 2019;85(4):574-581. doi:10.1002/ana.25434](https://sciwheel.com/work/bibliography/16034112)

[19.   Reddy S, Paramasivan NK, Sreedharan SE, Sukumaran S, Vinoda Thulaseedharan J, Sylaja PN. Association of 24 h Blood Pressure on Functional Outcome in Patients with Acute Ischemic Stroke Post Intravenous Thrombolysis. *Cerebrovasc Dis*. 2023;52(2):177-183. doi:10.1159/000526192](https://sciwheel.com/work/bibliography/16004781)
